# Identification of Hoiamide A as an Inducer of Oxidative and Endoplasmic Reticulum Secretory Pathway Stress

**DOI:** 10.1021/acs.jnatprod.6c00129

**Published:** 2026-05-04

**Authors:** Sophia E. Bonar, Daphne R. Mattos, Xinhui Yu, George F. Neuhaus, Diaa T. A. Youssef, Lamiaa A. Shaala, William H. Gerwick, Kerry L. McPhail, Jane E. Ishmael

**Affiliations:** ‡ Department of Pharmaceutical Sciences, College of Pharmacy, 2694 Oregon State University, Corvallis, Oregon 97331, United States; § Department of Natural Products, Faculty of Pharmacy, King Abdulaziz University, Jeddah 21589, Kingdom of Saudi Arabia; ∥ King Fahd Medical Research Center, King Abdulaziz University, Jeddah 21589, Kingdom of Saudi Arabia; ⊥ Department of Pharmacognosy, Faculty of Pharmacy, 68831 Suez Canal University, Ismailia 41522, Egypt; # Suez Canal University Hospitals, Suez Canal University, Ismailia 41522, Egypt; ∇ Center for Marine Biotechnology and Biomedicine, Scripps Institution of Oceanography, 7060 University of California, San Diego, La Jolla, California 92093, United States; ○ Skaggs School of Pharmacy and Pharmaceutical Sciences, University of California, San Diego, La Jolla, California 92093, United States

## Abstract

The endoplasmic reticulum (ER) to Golgi secretory compartment of eukaryotic cells is highly sensitive to changes in intracellular homeostasis. Using a primary screening assay that monitors the function of this pathway, we prioritized a cyanobacterial extract from the Red Sea that decreased secretion of a bioluminescent reporter, *Gaussia* luciferase (GLuc), in living cells. A comparison of LCMS2 data against the GNPS database revealed a match for macrocyclic depsipeptide hoiamide A (**1**). Biological testing confirmed the ability of **1** to induce a mixed, non-lethal stress response in human U87-MG glioblastoma cells; analysis of stress markers by qRT-PCR revealed early upregulation of superoxide dismutase 1 (SOD1) and C/EBP-homologous protein (CHOP) relative to the control. Co-treatment of cells with **1** (100 nM to 3 μM) and antioxidant *N*-acetylcysteine afforded full protection from **1**-induced decreases in GLuc secretion. We report that terminally differentiated human SH-SY5Y neuroblastoma cells, with a neuron-like phenotype, are highly sensitive to nanomolar concentrations of **1**, whereas undifferentiated cells remained viable at 3 μM. These results expand the known biology of hoiamides and suggest that neurotoxic potential of **1** is likely due to an inherent failure of neurons to adapt to the loss of redox homeostasis and sustained ER stress.

## Graphical abstract

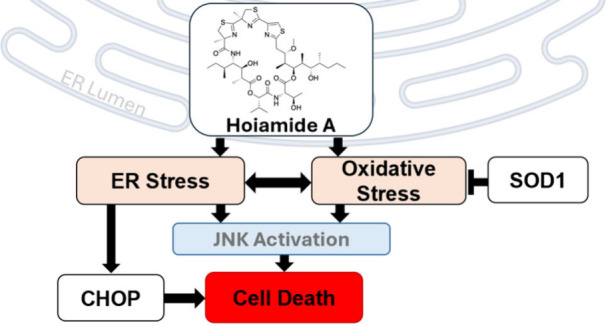

The conventional endoplasmic reticulum (ER) to Golgi secretory pathway is responsible for the biogenesis and maturation of approximately 30% of the human proteome and represents the route by which most soluble and membrane-bound proteins are trafficked to their final location at endomembranes, the surface, or outside the cell.^1,2^ Following import of nascent proteins into the ER lumen, or ER membrane, normal mammalian cells are equipped with multiple sequential mechanisms to ensure correct folding and assembly of proteins in the early secretory pathway before transport to the Golgi.^3^ Protein folding in the ER is carefully regulated in that any increase or accumulation of misfolded proteins triggers an unfolded protein response (UPR), designed to relieve acute ER stress and either restore proteostasis or actively induce pro-death signaling if ER stress is prolonged.^3–6^ It is well recognized that individual cell types vary widely in their ability to adapt to increase protein folding capacity and maintain ER proteostasis if physiological demands are increased.^5,6^ For example, the insulin-producing β-cells of the pancreas have an extensive ER network and high secretory capacity, yet are also vulnerable to proteotoxic ER stress that has long been linked to the pathogenesis of diabetes.^7,8^ Chronic ER stress, dysregulation of the UPR and loss of proteostasis is also well recognized in other human diseases, including neurodegenerative conditions, associated with intracellular protein accumulation, and tumor-supportive stress signaling in multiple cancer cell types.^8–10^

Natural products that induce site-specific changes in the function of the secretory pathway have become valuable reference compounds to probe the complex signaling events that regulate protein biosynthesis and maintenance of homeostasis in eukaryotic cells.^11,12^ The small molecule cycloheximide, from *Streptomyces griseus*, is just one of several specialized metabolites that target protein biogenesis at the level of the translating ribosome and is therefore commonly used by biologists as a standard chemical inhibitor of total protein synthesis.^13,14^ This mechanism of action can be distinguished from other major classes of natural products that act downstream of this process, after the translating ribosome has docked with the ER membrane, to block the biosynthesis of only those proteins that undergo cotranslational translocation into the ER via the Sec61 translocon channel.^12^ Cycloheximide was in fact used as a critical control in target validation of the more recently discovered natural product Sec61 inhibitors, ipomoeassin F and coibamide A, where pulse-labeling experiments in cultured cells revealed selective inhibition of only nascent glycosylated and secreted protein fractions while protein biogenesis in other subcellular compartments was preserved.^15,16^ Natural Sec61 translocon inhibitors can be further distinguished as they induce an atypical ER stress response,^17–20^ that is unlike classic chemical inducers of ER stress, such as thapsigargin, tunicamycin, or brefeldin A. The latter represent a class of different toxic specialized metabolites that have evolved to disrupt the maturation and/or trafficking of secretory pathway proteins after the nascent polypeptide has been imported into the ER.^12,21,22^

Proteins originating in the secretory pathway have historically been a rich source of druggable targets for the treatment of human disease, with an estimated 68% of FDA-approved drugs targeting a transmembrane or secreted protein.^1,23^ In our search for new pharmacological modulators of this pathway, we utilized a luciferase-based reporter assay to screen a collection of cyanobacterial extracts for their ability to elicit ER stress in living cells. As reported previously by the Tannous laboratory, human cells can be engineered to express a humanized form of the naturally secreted enzyme *Gaussia* luciferase (GLuc) for use as a sensitive screening tool to monitor protein homeostasis and ER stress in the secretory compartment.^24–26^ Under normal conditions, nascent secretory pathway proteins, including GLuc, are trafficked through the secretory compartment, but under stressed conditions unfolded proteins accumulate in the ER and GLuc secretion is reduced.^4,25^ This primary screen was successfully used as a cell viability assay to identify obtusaquinone from the *Dalbergia retusa* tree, as a potent inducer of cytotoxic reactive oxygen species (ROS) that, in advanced testing, has also shown *in vivo* efficacy in several mouse models of breast cancer and glioblastoma.^27,28^ Secretion of native GLuc into the extracellular space is critically dependent upon the presence of a N-terminal signal peptide, consistent with a mechanism by which nascent GLuc is cotranslationally translocated into the ER via the Sec61 translocon channel.^24^ Thus, we have established that direct inhibitors of the Sec61 translocon channel induce early, concentration-dependent decreases in GLuc expression in the cell culture medium of viable cells using this screening platform. Crucially, early changes in GLuc expression can be temporally uncoupled from basic biochemical and morphological features of regulated cell death in the adherent cells.^29^ In turn, this ability to separate physiological function from cell death facilitated efficient pharmacologic profiling of synthetic diastereomers and analogues of the natural product Sec61 inhibitor coibamide A.^30,31^ The high sensitivity of the GLuc assay as a screening tool to report on the status of the secretory pathway also enables detection of new bioactivity in natural product samples that are not overtly cytotoxic to established cancer cell lines in standard end point viability assays. Such was the case with amatyemide B, a recently discovered cyclic octadepsipeptide from South African stromatolites that induced oxidative ER stress in human cancer cells at low micromolar concentrations.^32^ Here, we report the Global Natural Product Social Molecular Networking (GNPS)-guided rediscovery of hoiamide A (**1**) in bioactive extracts prepared from field-collected samples of Red Sea cyanobacteria using the same functional screening assay.^33^ Follow-on studies with authentic hoiamide A (**1**), a cyclic depsipeptide with a mixed peptide-polyketide structure,^34^ demonstrated the ability of compound **1** to act as a pharmacologic disrupter of intracellular homeostasis resulting in oxidative and ER stress.
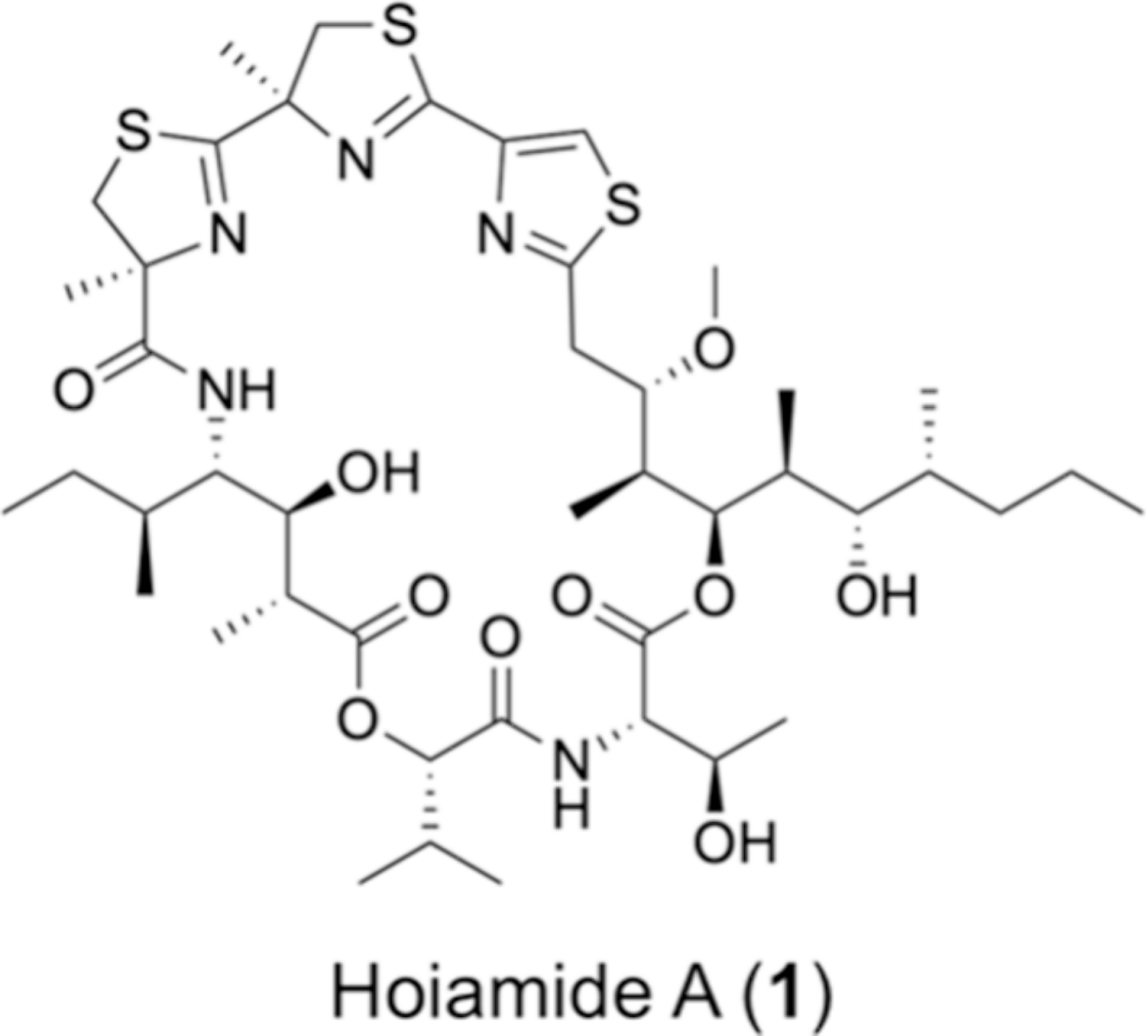


## Results and Discussion

### Bioassay-Guided Identification of Hoiamide A as an Inducer of ER Stress

A mixed assemblage of cyanobacteria was collected by hand in shallow water at two different locations: in the Suez Canal at Ismailia, Egypt (DY-70) and, at the shore of the Nabq mangrove forest on the Gulf of Aqaba, Red Sea (DY-71). Seventeen chromatographic fractions (DY-70-3 to DY-70-10 and DY-71-2 to DY-71-10) from the two organic extracts obtained from the field-collected material were screened simultaneously in three independent phenotypic assays for their ability to (1) inhibit GLuc bioluminescent activity in cell culture medium collected 16 and 24 h after exposure of human U87-MG glioblastoma cells engineered to express GLuc (U87-GLuc), (2) inhibit the viability of wild-type human U87-MG glioblastoma cells at 48 h and, (3) inhibit the viability of human HeLa cervical cancer cells at 72 h. Fractions DY-71-7 and DY-71-8, eluted with hexanes/EtOAc (30:70) and EtOAc (100%) over normal phase silica, respectively, were prioritized from these primary screens and flagged for further evaluation. At 16 and 24 h after exposure, both fractions induced a significant decrease (>50%) in GLuc expression in aliquots of the cell-free conditioned medium, relative to control (0.1% DMSO), without significant change in viability of the reporter cells (Figure 1A). These fractions (DY-71-7 and DY-71-8) also induced a modest decrease (∼30%) in the viability of wild-type U87-MG glioblastoma cells at 48 h (Figure 1B) and no change in the viability of human HeLa cervical cancer cells at 72 h (Figure 1C).

**Figure 1:**
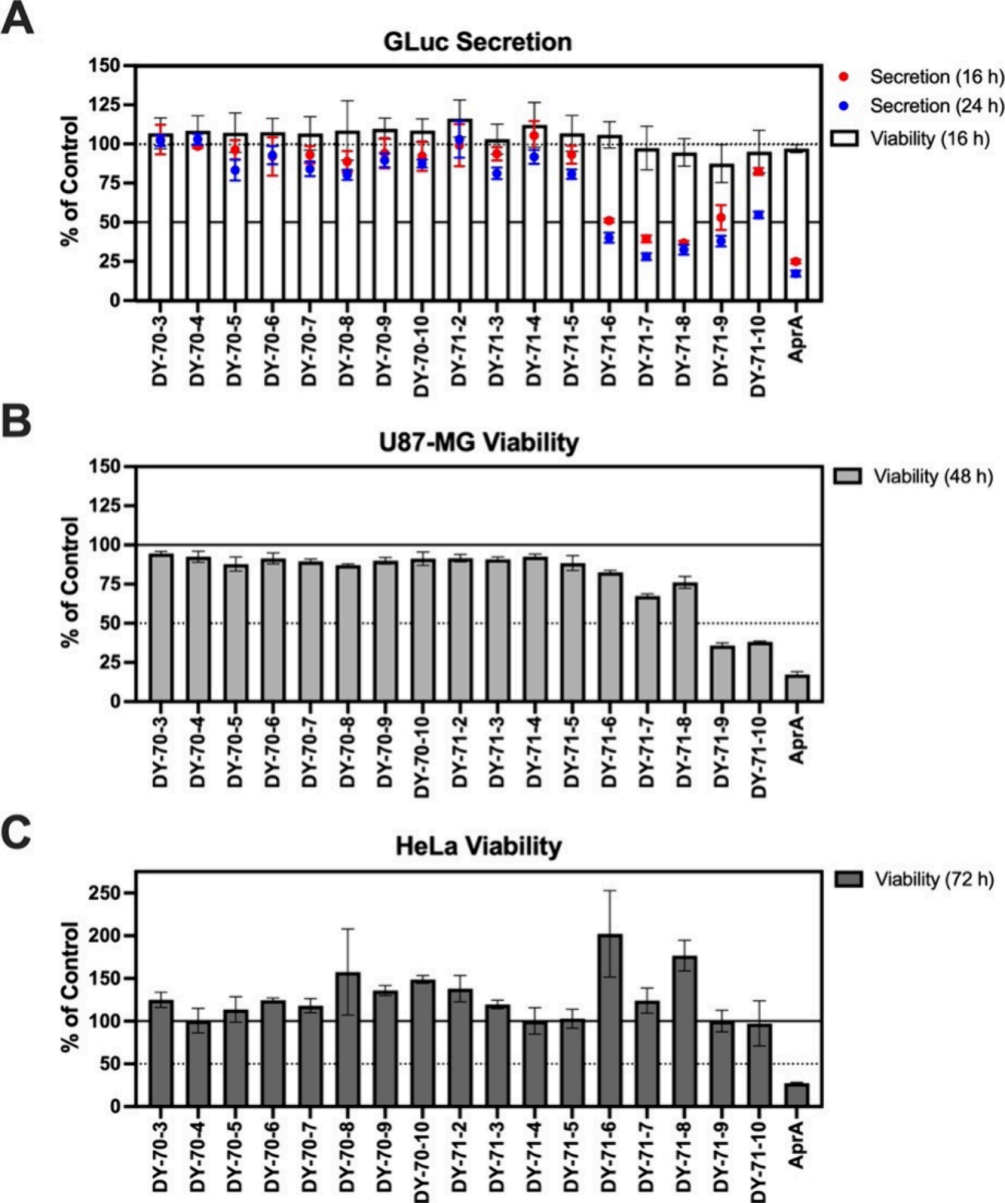
Primary screening results for Suez Canal and Red Sea cyanobacterial extracts. Seventeen first tier fractions from the Suez Canal (DY-70) or Red Sea (DY-71) were screened in three different cell-based assays for potential to modulate: (A) expression of secreted GLuc at 16 h (red circle) and 24 h (blue circle) in the culture medium, and the viability (white column) of adherent U87-GLuc reporter cells at 16 h, (B) cytotoxicity (pale gray column) to human wild-type U87-MG glioblastoma cells at 48 h, and (C) cytotoxicity (dark gray column) to human wild-type HeLa cervical cancer cells at 72 h. Cells were treated simultaneously (100 μg/mL; *n* = 3 wells per assay) and returned to the incubator until the assay end point (16, 24, 48, or 72 h), apratoxin A (30 nM) served as a positive control on all plates. Data represents the results of the full primary screen in three cell lines that was duplicated. All values are expressed as % change relative to vehicle (0.1% DMSO)-treated cells.

Based on the observed time-sensitive decreases in GLuc activity, coupled with the potential for differential cell-type sensitivity, fractions DY-71-7 and DY-71-8 from the Gulf of Aqaba collection site were evaluated against the GNPS spectral libraries to allow automated dereplication of potentially known compounds in these samples.^35^ Specifically, natural product inhibitors of the Sec61 translocon channel induce a similar pattern of GLuc inhibition in living cells at 18h.^29^ We have previously reported the isolation of several apratoxins from a Red Sea *Moorea producens*,^36^ and while the presence of an apratoxin-like molecule in DY-71-8 could not be ruled out, none of the LCMS^2^ spectra collected for DY-71-7 matched the deposited apratoxin A or coibamide A spectra. Rather, these MS^2^ data included spectra that aligned closely with the GNPS Silver reference spectrum for hoiamide A. Hoiamide A is a cyclic depsipeptide first isolated from a mixed extract of two cyanobacteria (*Moorea producens* and *Phormidium gracile*) collected by SCUBA in Hoia Bay, Papua New Guinea.^34^ The hoiamide A-like compound was identified in the DY-71-7 extract with a cosine score of 0.8, where a cosine score of 0 is totally dissimilar and 1 indicates a perfect match.^35^ Due to the relatively high cosine score and high quality of the hoiamide A reference spectra in the GNPS database, we hypothesized that sample DY-17-7 may contain a hoiamide A-like compound.

The DY-71-7 fraction was further purified using solid-phase extraction (SPE), with a stepwise hexane:EtOAc:MeOH gradient over normal phase silica, to yield six fractions of increasing polarity (A–F). LCMS^1^ data were collected and interrogated for a mass feature matching the exact mass of hoiamide A (*m*/*z* 926.444). Fractions A–C all harbored a positive match and were further processed with reverse phase SPE to yield subfractions: A1-3, B1-2, and C1-3. ^1^H NMR spectra were acquired for these subfractions and analyzed for α-proton chemical shifts between 3 and 5 ppm. Signals corresponding to a peptide signature were identified in all the C subfractions (Figure S1), and thus high-performance liquid chromatography (HPLC) was used to purify the metabolite predicted to be hoiamide A for further chemical and biological screening. As compound **1** was originally isolated in 65% acetonitrile in water,^34^ this portion of a solvent gradient of 15% acetonitrile to 100% acetonitrile in water containing 1% formic acid was prioritized for further evaluation. LCMS data for the C1C subfraction revealed a single major constituent with associated mass *m*/*z* 926.4438 (Figure S2). Co-injection of the C1C fraction with authentic hoiamide A (**1**)^34^ confirmed that they possessed the same elution time (6.5 min) and mass values (C1C fraction = *m*/*z* 926.4438, authentic compound **1** = *m*/*z* 926.4445, Δppm 0.76), supporting the presence of **1** in Red Sea cyanobacterial extracts (Figure S3). LCMS^2^ data also showed closely matched fragmentation patterns identified by *m*/*z* and intensity in authentic **1** and the C1C subfraction (Figure 2 and Table S1) as well as similar isotopic patterns consistent with the sulfur atoms present in the sequential thiazolines and thiazoles in **1** ^34^ (Table S2). After these purification steps (methods included in the Supporting Information), further evaluation of the LCMS^2^ data with GNPS showed that the Red Sea extract and the authentic standard of **1** share a common mass feature in a classical molecular network (Figure S4), and the cosine score of the Red Sea sample relative to the authentic standard was 0.95. These results led to the reasonable conclusion that compound **1** was the major bioactive component flagged as a hit after phenotypic screening of the Red Sea (DY-71) cyanobacterial extract library (Figure 1).

**Figure 2:**
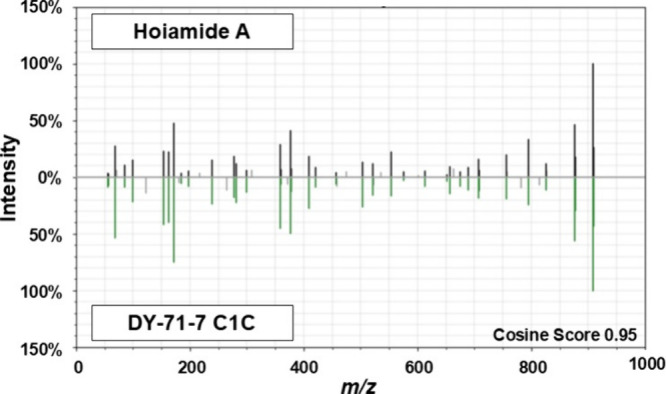
LCMS^2^ mirror plot of the hoiamide A reference and the DY-71-7 C1C experimental spectrum. Comparison of fragmentation patterns for MS^2^ data collected from dual injection of the hoiamide A analytical reference (gray; upper spectrum) and DY-71-7 C1C experimental sample (green; lower spectrum). Samples were dissolved in LCMS-grade methanol, filtered and injected on an Agilent 1260 infinity II LC coupled to a 6545 QToF MS. Spectral features were analyzed using the Global Natural Products Social Molecular Networking (GNPS) database and the mirror plot created using the GNPS metabolomics USI feature. Comparison of the hoiamide A analytical reference and DY-71-7 C1C experimental reference resulted in a cosine score of 0.95.

### Differentiated SH-SY5Y Cells Acquire Increased Sensitivity to Hoiamide A

As insufficient source material was available to pursue scale-up purification of hoiamide A from the DY-71-7 fraction, we relied on previously isolated authentic **1** ^34^ for further biological testing. The hoiamide family is comprised of four complex marine natural products; hoiamides A (**1**) and B are cyclic depsipeptides, whereas hoiamides C and D are linear molecules.^34,37,38^ Hoiamide A (**1**) is constructed from highly functionalized/unusual amino acids and fatty-acid-like polyketide chains stitched together through amide/ester bonds and the formation of cysteine-derived heterocycles. The macrocyclic hoiamide chemotypes each have a distinct pharmacological profile and although both compounds showed weak cytotoxicity to human NCI-H460 nonsmall cell lung cancer cells at micromolar concentrations, hoiamide A (**1**) was more toxic (IC_50_ = 2.1 μM) than hoiamide B to mouse Neuro-2A neuroblastoma cells and was found to be surprisingly toxic to primary neocortical neurons established from embryonic mouse cortex.^37,39,40^ In the study by Cao and coauthors, pure neuronal cultures maintained for 8 to 10 days *in vitro* showed characteristics of both apoptosis and necrosis within hours of exposure to nanomolar concentrations of hoiamide A.^39^ This complex pattern of regulated cell death was not attributed to the known actions of **1**: as a weak partial agonist at voltage-gated sodium channels, or as an inhibitor of the spontaneous calcium oscillations that can be recorded from mature cultures of primary neurons.^34,41^ However, a pharmacological inhibitor of Jun NH_2_-terminal kinase (JNK), SP600125, was shown to afford statistically significant protection against hoiamide A-induced neurotoxicity by two independent measures of cell death and in a pattern that was not observed with small molecule inhibitors of other mitogen activated protein (MAP) kinases.^39^ The potential for hoiamide A to activate endogenous JNK signaling in eukaryotic cells was further established when immunoblot analysis of lysates from cultured neurons showed time-sensitive increases in JNK phosphorylation within hours of exposure to **1**.^39^

Given the marked differential sensitivity of primary neurons to **1**, relative to all immortalized cancer cell lines tested to date,^34,37,39^ we first compared the cytotoxic potential of **1** against undifferentiated and differentiated human SH-SY5Y neuroblastoma cells. The SH-SY5Y *in vitro* cell culture model has been widely used by the neuroscience community as a basic research tool for early stage drug discovery and the study of neurodegenerative disease.^42,43^ In the undifferentiated state, SH-SY5Y cells maintain a neuroblast phenotype and continue to proliferate under standard growth conditions, however the same cells can also be induced to differentiate to a neuron-like phenotype that gradually stops proliferating and acquires molecular and morphological characteristics of neurons.^42–44^ For these studies, SH-SY5Y cells were terminally differentiated over 11 days by sequential treatment with retinoic acid (10 μM) for 5 days, followed by brain-derived neurotrophic factor (BDNF; 50 ng/mL) for a further 5 days, using a method optimized by the O’Carroll laboratory.^44^ Immunoblot analysis of whole-cell lysates prepared from differentiated and undifferentiated SH-SY5Y cells revealed expression of βIII-tubulin only in differentiated cells (Figure S5), indicating that these cultures had acquired a neuron-specific marker of microtubules.^45^ For analysis of cell morphology, differentiated and undifferentiated SH-SY5Y cells were established on glass coverslips and treated with hoiamide A (1 μM) or vehicle (0.1% DMSO) for 24 h. Cells were then fixed and processed for immunocytochemistry of βIII-tubulin and α-tubulin with the same isoform-specific primary antibodies, appropriate secondary antibodies plus DAPI stain to visualize the cell nuclei (Figure 3A). Fluorescence microscopy revealed a predominantly cytoplasmic distribution of α-tubulin in both differentiated and undifferentiated SH-SY5Y cells (Figure 3Aa–d). However, the neurites of differentiated SH-SY5Y cells were well-developed only in vehicle-treated cells (Figure 3Ac) and were significantly diminished, with weak fluorescence intensity for both βIII-tubulin (Figure 3Ad) and α-tubulin (Figure 3Ah) in hoiamide-treated SH-SY5Y cells. Quantification of these images suggested that the weak fluorescence intensity may be attributed to a decrease in the total number of neurites associated with each cell body, however differences in the number of neurites/cell (Figure 3B) or length of neurites (Figure 3C) were not statistically significant. In a separate analysis, we exposed differentiated and undifferentiated SH-SY5Y cultures to increasing concentrations of hoiamide (100 nM to 3 μM), the fungal natural product brefeldin A (3 μM) or vehicle (0.1% DMSO) and assessed the viability of all cells using a standard end point assay at 96 h (Figure 3D). Under these conditions, undifferentiated SH-SY5Y cells remained viable in the presence of up to 3 μM of **1**, whereas differentiated SH-SY5Y cells showed a statistically significant loss of viability after exposure to 300 nM hoiamide A (Figure 3D). This differential pattern of sensitivity to **1** was not observed in cells exposed to brefeldin A, in that both differentiated and undifferentiated SH-SY5Y were equally vulnerable to pharmacologic inhibition of ER to Golgi traffic in the secretory pathway (Figure 3D). In agreement with the previous study by the Murray laboratory,^39^ these results demonstrate the increased sensitivity of cells with a neuronal phenotype to hoiamide A. Using the same time of exposure (24 h) for analysis of neurite morphology, we observed a loss of neurite-associated fluorescence in terminally differentiated SH-SY5Y cells after exposure to **1** (Figure 3A–C), whereas in the previous study the neurites of primary neocortical neurons failed to grow and develop normally in the presence of **1**.^39^ This loss of neurite complexity preceded hoiamide-induced loss of metabolic function in the SH-SY5Y neuroblastoma cell culture model (Figure 3D), whereas multiple damage-associated molecular patterns (DAMPs) of cell death were observed in primary neurons within hours of exposure to **1**.^39^ Together our results extend and support the previous reclassification of hoiamide A as a neurotoxin with marked cell-type specificity.

**Figure 3:**
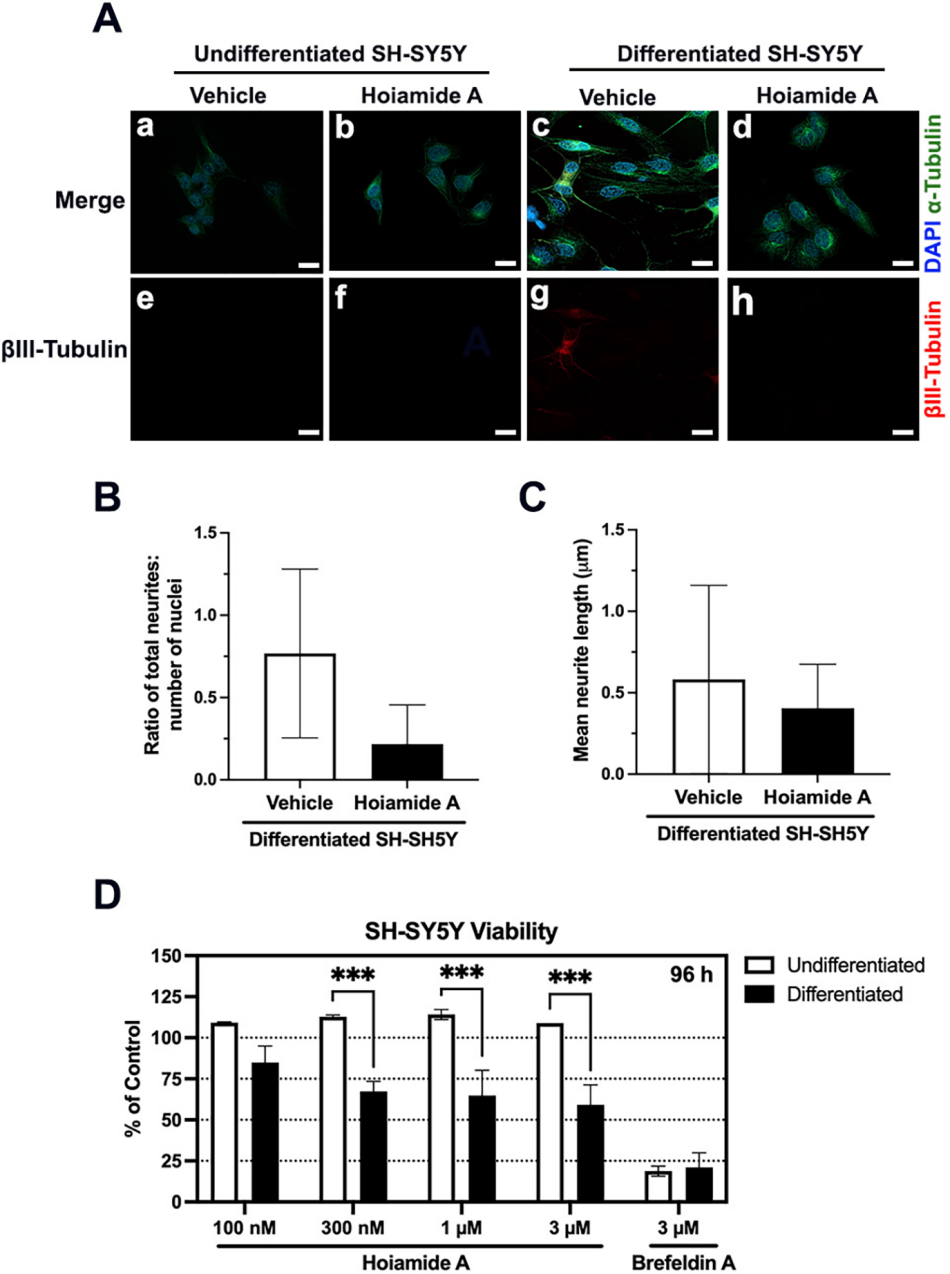
Terminally differentiated SH-SY5Y neuroblastoma cells are more sensitive to hoiamide A-induced cytotoxicity than undifferentiated cells. Comparison of the cytotoxic potential of hoiamide A to undifferentiated and differentiated, neuron-like, human SH-SY5Y neuroblastoma cells relative to vehicle-treated cells. SH-SY5Y cells were terminally differentiated over 11 days by sequential treatment with retinoic acid (10 μM) for 5 days, followed by brain-derived neurotrophic factor (BDNF; 50 ng/mL) for 5 days. (A) Immunocytochemistry of α-tubulin (green), DAPI (blue) and βIII-tubulin (red) in undifferentiated and differentiated SH-SY5Y cells treated with hoiamide A (1 μM) or vehicle (0.1% DMSO) for 24 h. Images are from an experiment that was replicated three times. Scale bar = 20 μm. (B) Quantification of neurites and (C) quantification of neurite length (μm) in terminally differentiated SH-SY5Y cells in response to hoiamide A (1 μM) or vehicle (0.1% DMSO) for 24 h. (D) Viability of undifferentiated (white) and differentiated (black) SH-SY5Y cells treated with hoiamide A (100 nM - 3 μM), brefeldin A (3 μM), or vehicle (0.1% DMSO) for 96 h. Histogram represents viability relative to vehicle-control; *n* = 3 wells/treatment from two biological replicates. Statistical significance of change is (∗∗∗) *p* < 0.005.

### Hoiamide A Triggers an Intracellular Antioxidant Response

Given the potential for hoiamide A to induce ER stress in human glioblastoma cells (Figure 1A), and early phosphorylation of JNK in neurons,^39^ we surveyed a panel of ER and oxidative cell stress markers for changes in gene expression. As a major sensor of intracellular stress, JNK can be activated through the inositol-requiring enzyme 1 (IRE1) branch of the UPR^46–48^ and, also by the loss of redox homeostasis.^49^ For these studies, wild-type human U87-MG glioblastoma cells were treated with 1 (3 μM) or vehicle (0.1% DMSO) for 4 h and the relative expression of several stress-induced transcripts was measured by real-time quantitative PCR (qPCR). Hoiamide A (**1**) failed to induce a robust ER stress response under these conditions, in that when expression of the genes encoding glucose-regulated protein of 78 kDa (GRP78), C/EBP-homologous protein (CHOP) and spliced X box-binding protein1 (sXBP1) were compared, only CHOP showed a statistically significant increase in gene expression relative to vehicle-treated cells (Figure 4A). However, as CHOP is considered a sensitive biomarker of direct or indirect ER stress,^48,50^ we analyzed expression of hypoxia-inducible factor 1 subunit α (HIF1A), nuclear factor erythroid 2 like factor (NRF2), glutathione-disulfide reductase (GSR), glutathione peroxidase 1 (GPX1), glutathione peroxidase 4 (GPX4) and superoxide dismutase 1 (SOD1) using the same experimental conditions. Of these six genes, only SOD1 showed a statistically significant up-regulation in hoiamide A-treated cells relative to vehicle-treated control cells (Figure 4B).

**Figure 4:**
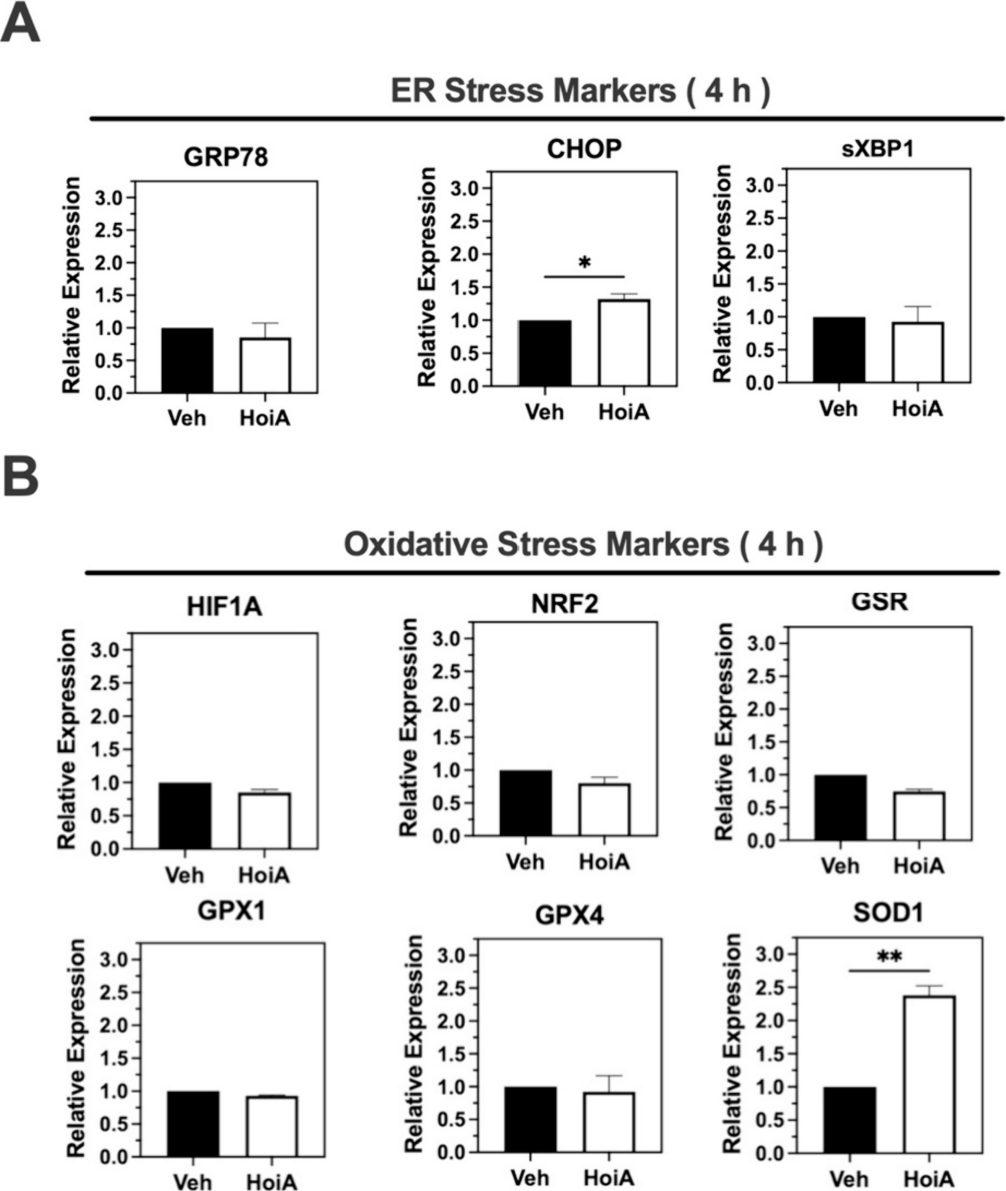
Hoiamide A induces a specific stress signature. Relative mRNA expression of (A) ER markers (GRP78/BiP, CHOP, and xSBP1) and (B) oxidative stress markers (HIF1A, NRF2, GSR, GPX1, GPX4, and SOD1) in human U87-MG glioblastoma cells treated with hoiamide A (3 μM) relative to control (0.1% DMSO). Cells were treated for 4 h and transcripts were measured by qPCR normalized to β-tubulin. Data are expressed as fold-change, compared to vehicle, from three biological replicates. Statistical significance of change is (∗) *p* < 0.05 and (∗∗) *p* < 0.01.

SOD1 is an intracellular enzyme that is induced primarily in response to increases in superoxide, as part of a wider antioxidant and reactive oxygen species (ROS)-scavenging network.^51^ To evaluate the potential contribution of intracellular ROS to hoiamide A-induced ER stress, we used the GLuc-U87 reporter cell line to assess ER secretory function in response to increasing concentrations of **1** (100 nM to 3 μM), the cyanobacterial parent fraction (100 μg/mL), apratoxin A (300 nM) or vehicle (0.1% DMSO) in the presence or absence of *N*-acetylcysteine (NAC). In the absence of NAC, **1** (100 nM to 3 μM), DY-71-7 and apratoxin A all induced decreases in extracellular GLuc expression relative to vehicle-treated cells at 18h (Figure 5A). However, the antioxidant NAC (3 mM) afforded a statistically significant rescue of GLuc expression in the cell culture medium at all concentrations of **1** tested (Figure 5A), in a pattern that was also observed with rotenone (1 μM), a control inducer of intracellular ROS (Figure S6). Modest rescue of GLuc expression was also observed in GLuc-U87 cells exposed to the parent DY-71-7 fraction plus NAC, but not in cells exposed to apratoxin A, a direct Sec61 inhibitor of protein import into the ER (Figure 5A). As anticipated, hoiamide A (100 nM to 3 μM) and apratoxin A (300 nM) induced no significant change in the viability of GLuc-U87 cells at 18h (Figure 5B), whereas rotenone (1 μM) was overtly cytotoxic and thus NAC restored both GLuc expression and cell viability at 18 h relative to rotenone treatment alone (Figure S6). In summary, these results demonstrate that **1** induces a relatively early stress response in U87-MG glioblastoma cells, in that mRNAs encoding SOD1 and CHOP were increased within hours of exposure. Thus, from our analysis of the secretory function of living U87-MG cells, we propose a mechanism whereby **1** disrupts the internal redox status of the cell to an extent that is sufficient to directly, or indirectly, induce stress in the ER. In U87-MG glioblastoma cells, hoiamide A-induced stress was sufficient to reduce GLuc secretion as an adaptive response to **1** but, as with undifferentiated SH-SY5Y cells, this was not sufficient to trigger cell death.

**Figure 5:**
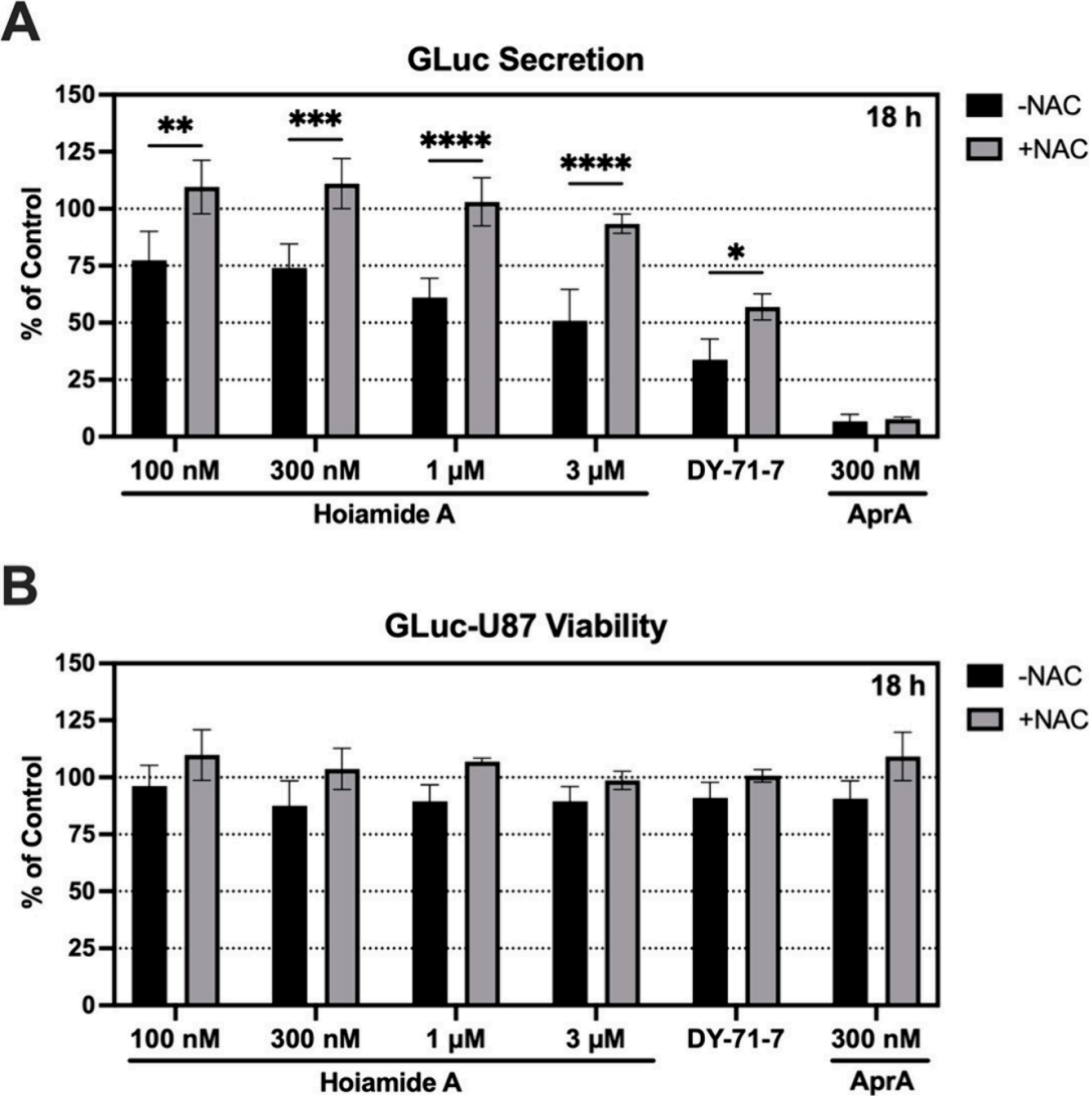
Antioxidant *N*-acetylcysteine (NAC) rescues hoiamide A-induced decreases in ER secretory function. Comparative analysis of the secretory function of viable hoiamide A-treated cells in the presence or absence of *N*-acetylcysteine (NAC). The secretory function (A) and viability (B) of human U87-MG glioblastoma cells expressing *Gaussia* luciferase (GLuc) was assessed 18 h after treatment with hoiamide A (100 nM–3 μM), cyanobacterial extract DY-71-7 (100 μg/mL), apratoxin A (300 nM), or vehicle (0.1% DMSO) in the presence and absence of NAC (3 mM). Data are expressed as fold-change compared to vehicle (100%). Bars represent *n* = 3 wells/treatment from three biological replicates. Statistical significance of change is (∗) *p* < 0.05, (∗∗) *p* < 0.01, (∗∗∗) *p* < 0.005, and (∗∗∗∗) *p* < 0.001.

## Conclusion

The cyclic depsipeptide hoiamide A (**1**) was detected in samples of marine cyanobacteria collected in the Red Sea. Bioactive fractions were initially prioritized as hits in a phenotypic screening assay that reports on the functional status of the conventional ER secretory pathway and later identified through comparative analysis of LCMS^2^ data for the Red Sea sample relative to reference spectra for compound **1** in the GNPS database. The ability of **1** to induce cell stress and impair ER secretory function in living cells was confirmed with authentic **1** isolated from marine cyanobacteria collected in Papua New Guinea. Although the direct cellular target and mechanism by which **1** induces intracellular stress is not yet known, our addition of SOD1 and CHOP to the pattern of cellular consequences that occur in response to **1** compliment and extend previous results in primary neocortical neurons, where activation of the stress-induced JNK pathway preceded cell death.^39^ Oxidative and ER stress are highly related, in that the ER is both sensitive to changes in redox status and is, itself, a major site of ROS production as a normal consequence of protein biosynthesis and folding for approximately one-third of the proteome.^52,53^ In light of a published route for total chemical synthesis,^54^ and having confirmed that **1** elicits relatively weak responses, or is inactive, against most established human cancer lines, future mechanistic studies of target engagement and activation of JNK-CHOP stress and death signaling by **1** would ideally be pursued using primary neurons or an equivalent neuron-like cell model.

## Experimental Section

### General Experimental Procedures

#### Chemicals, Reagents and Antibodies

The isolation and purification of authentic hoiamide A and apratoxin A has been described previously.^34,36^ Each natural product was reconstituted in 100% cell culture grade DMSO Millipore-Sigma (Burlington, MA, U.S.A.), aliquoted and stored as a concentrated (mM) stock solutions in amber borosilicate glass vials at −20 °C for use in biological studies. Brefeldin A was purchased from Cell Signaling Technology, Inc. (Davers, MA, U.S.A.) and *N*-acetyl cysteine (NAC) was from Calbiochem (San Diego, CA, U.S.A.), both were reconstituted in 100% DMSO and stored at −20 °C. Coelenterazine was from Promega Corporation (Madison, WI, U.S.A.) and was reconstituted in methanol. The α-tubulin rabbit polyclonal (2144) and βIII-tubulin mouse monoclonal (4466S) primary antibodies, plus HRP-conjugated secondary antibodies were from Cell Signaling Technology, Inc. Secondary antibodies conjugated to the cyanine fluorophores Cy3 and Cy5 were from Jackson ImmunoResearch Laboratories (West Grove, PA, U.S.A., 711-165-152). All general laboratory reagents were from VWR International (Radnor, PA, U.S.A.).

#### Sample Collection and Extraction for Primary Screen

Mixed assemblages of cyanobacteria were collected by hand at (1) a depth of 0.5 m in the Suez Canal at Ismailia, Egypt (DY-70, an encrusting greenish brown mass) and (2) a depth of 1.5 m at the shore of the Nabq mangrove forest on the Gulf of Aqaba, Red Sea (DY-71, predominantly *Moorea producens* based on morphology) and immediately kept on ice and stored at −20 °C until extraction. Both cyanobacterial samples are deposited in the Red Sea collection at Suez Canal University using their respective collection codes (DY70 and DY71). The cyanobacterial material (2.3 kg, wet weight for each collection) was extracted with CH_2_Cl_2_/MeOH (1:1) and the resulting extract was partitioned on a normal phase (SiO_2_) vacuum liquid chromatography (VLC) column using hexane–EtOAc–MeOH gradients to give 17 main fractions. For phenotypic screening, dry material (100 μg) was resuspended in cell-culture grade DMSO (20 μL) and each coded sample systematically mapped to an individual well in a 96-well V-bottom compound plate with appropriate controls. On the day of the experiment, samples were delivered to each of three prepared cell culture plates (final concentration = 100 μg/mL) for each assay using an automated liquid handling system and Caliper pin tool (resulting in *n* = 3 technical replicates). Screening plates were immediately returned to the incubator and maintained under standard conditions (37 °C in an atmosphere of 5% CO_2_) until the end point of each assay. Hits were prioritized after replication of the full screen (*N* = 6 points for each assay).

#### Mammalian Cell Culture

Human U87-MG glioblastoma, HeLa cervical and SH-SY5Y neuroblastoma cells were from the American Type Culture Collection (ATCC, Manasses, VA, U.S.A.). The method for generation of the reporter cell line (GLuc-U87) from wild-type U87-MG cells has been described previously.^25^ HeLa, GLuc-U87 and U87-MG glioblastoma cells were cultured in minimum essential medium (MEM) with Earl’s salts and l-glutamine (Corning Life Sciences), supplemented with 10% fetal bovine serum (FBS, Hyclone, Logan, UT, U.S.A.) and 100 U/mL penicillin and 100 mg/mL streptomycin (1% penicillin//streptomycin). Undifferentiated SH-SY5Y cells were maintained in Dulbecco’s modified Eagle’s medium (DMEM) supplemented with 10% FBS and 1% penicillin/streptomycin. All cells were grown under standard conditions and maintained at 37 °C in an atmosphere of 5% CO_2_.

### *Gaussia* Luciferase (GLuc) Secretory Assay

GLuc-U87 cells were seeded at 3000 cells/well into 96-well plates and allowed to adhere overnight. The next day, seeding medium was replaced with complete medium containing extracts (primary screen), test compounds or vehicle (0.1% DMSO) and plates were returned to the incubator. At the end point, conditioned cell culture medium (20 μL) was removed from each well and transferred to 96-well white-walled plates. Expression of GLuc was assessed by the addition of substrate coelenterazine into each well (final concentration of 1.2 μM), and luminescent signals were measured using a multimode microplate reader (Biotek Synergy HT) with Gen5 software and compared across conditions (3 s wait, 0.5 s integration time following coelenterazine injection).

### Cell Viability Assays

Wild-type U87-MG (3000 cells/well), HeLa cells (2000 cells/well), or SH-SY5Y cells (2500 cells/well) were seeded into 96-well plates, allowed to adhere overnight before treatment with extracts (primary screen), test compounds or vehicle (0.1% DMSO). The viability of all cells, including adherent GLuc-U87 cells used for monitoring cell secretion, was assessed at the end point of each assay using a CellTiter-Glo Luminescent Cell Viability assay designed to detect ATP in metabolically active cells (Promega Corp.). Luminescent signals were measured using the same Biotek Synergy HT multimode microplate reader.

### Differentiation of SH-SY5Y Cells

SH-SY5Y neuroblastoma cells were differentiated to a neuronal-like phenotype according to the method described by Dravid et al.^44^ Briefly, low-passage undifferentiated SH-SY5Y cells were seeded at 2500 cells/well into 96-well plates that had been pretreated with Geltrex basement matrix (Thermo Fisher Scientific, Inc.) and allowed to adhere overnight. On day 1, the cell culture medium (DMEM supplemented with 10% FBS and 1% penicillin/streptomycin) was replaced with same DMEM medium, as above, containing 10 μM retinoic acid (phase 1) for 5 days. On day 6, the cell culture medium was replaced with the same medium (phase 2) containing 50 ng/mL brain-derived neurotrophic factor (BDNF) to promote terminal differentiation by day 11. After day 11 differentiated cells were used alongside undifferentiated parental SH-SY5Y cells that were maintained under standard conditions during the time required for differentiation.

### Western Blot Analysis

Differentiated and undifferentiated SH-SY5Y cells were lysed as described previously.^55^ Whole-cell lysates were adjusted for protein content using a bicinchoninic acid assay (Pierce) and mixed with standard Laemmli sample buffer. Proteins were separated by SDS–PAGE, immobilized onto PVDF membranes and then blocked in 5% (w/v) nonfat dry milk in 50 mM Tris–HCl, pH 7.4 containing 150 mM NaCl (TBS) plus 0.1% Tween-20 (TBS-Tween). Membranes were incubated overnight (at 4 °C), with gentle rotation, in the primary antibody, rinsed in TBS–Tween (3 × 5 min) then incubated with the appropriate HRP-conjugated secondary antibodies for 1 h at RT. Membranes were rinsed again in TBS–Tween (3 × 5 min) at RT and proteins revealed by chemiluminescence (Amersham ECL reagent, GE Healthcare, Chicago, IL, U.S.A.). Images were captured using an iBright CL1500 image analysis system (Thermo Fisher Scientific).

### Immunocytochemistry

Differentiated and undifferentiated SH-SY5Y cells were established on glass coverslips, washed twice in phosphate-buffered saline (PBS) and fixed with 4% paraformaldehyde (PFA) for 20 min at RT. Cells were then washed twice in PBS and the PFA quenched with complete medium for 10 min at RT. Following two additional washes in PBS, permeabilized cells were incubated with 10% Triton X-100. Cells were then washed twice with PBS and blocked with 1% bovine serum albumin (BSA) in PBS (BSA/PBS) for 15 min. Cells were incubated for 1 h with anti-α-tubulin and βIII-tubulin antibodies, each diluted in 1:500 in BSA/PBS. The coverslips were washed in BSA/PBS (3 × 10 min) and then incubated with Cy3 and Cy5 fluorophore-conjugated secondary antibodies, each diluted 1:500 BSA/PBS for 30 min at RT. Coverslips were washed again (3 × 10 min) in BSA/PBS then mounted onto glass microscope slides with ProLong Gold Antifade with DNA stain DAPI (Thermo Fisher Scientific). Slides were observed using fluorescence microscopy and images captured using a ZEISS microscope using AxioVision software.

### Quantitative Real-Time PCR (qPCR)

Wild-type U87-MG glioblastoma cells were seeded at 100 000 cells/well in 6-well plates and allowed to adhere overnight. The next day cells were treated with hoiamide A or vehicle (0.1% DMSO) and, at the end point of the assay, RNA was extracted from each well using a RNeasy kit (Qiagen, Hilden, Germany) and transcribed to cDNA using an iScript cDNA synthesis kit (Bio-Rad, Hercules, CA, U.S.A.). The purity and quantity of all RNA and cDNA samples was tested using a NanoDrop spectrophotometer (Thermo Fisher Scientific). For qPCR, reaction mixtures contained 100 ng of cDNA, 400 nM of the forward and reverse primer for each gene of interest and 5 μL iTaq Universal SYBR Green Supermix (Bio-Rad) in a final volume of 10 μL. Fluorescent signals were detected using a 7500 Real Time PCR system (Applied Biosciences) using integral software to record the amplification plot, and threshold cycle (*C*_t_), for each transcript. All qPCR runs included three technical replicates with human β-actin used as an internal normalization control. The relative change in each gene of interest was subsequently calculated relative to the expression of the target gene in the vehicle control-treated cultures.^56^ All primers were purchased from Integrated DNA Technologies (San Diego, CA, U.S.A.); full primer sequences are provided in Table S3.

### Data Analysis

Concentration–response relationships were analyzed by nonlinear regression analysis fit to a logistic equation using GraphPad Prism Software version 10.0.02 (GraphPad Software, Inc., San Diego, CA, U.S.A.). The neurites of differentiated SH-SY5Y cells were quantified using ImageJ software (rsbweb.nih.gov/ij) from random fields of view per coverslip. Each whole DAPI-stained nucleus present in the field of view was considered one cell. For immunoblot analysis, signals were normalized to the intensity of α-tubulin and quantified using ImageJ software. Statistical significance of data was assessed using a one-way analysis of variance (ANOVA) followed by a Student’s *t* test comparing controls and treatment groups. *p* values less than 0.05 were considered significant.

## Supplementary Material



## Notes

Chemical characterization data, supporting biological figures, primer sequences, and additional experimental details (PDF)

## ◆

Department of Medicinal Chemistry, College of Pharmacy, University of Utah, Salt Lake City, Utah 84103, United States
