# Supplementary material for: Identification of Hoiamide A as an Inducer of Oxidative and Endoplasmic Reticulum Secretory Pathway Stress
Source: J Nat Prod. 2026 May 4;89(5):1513–23. doi: 10.1021/acs.jnatprod.6c00129 (PMC13200239; doi:10.1021/acs.jnatprod.6c00129)
Supplement: Supplementary file 1 [file np6c00129_si_001.pdf]

Supporting Information for

Identification of Hoiamide A as an Inducer of Oxidative and ER Secretory Pathway Stress.

Sophia E. Bonar [1,#], Daphne R. Mattos [1,#], Xinhui Yu [1], George F. Neuhaus [1], Diaa T. A. Youssef [2,3,4], Lamiaa A. Shaala [5], William H. Gerwick [6,7], Kerry L. McPhail [1,^,*] and Jane E. Ishmael [1,*]

[1] Department of Pharmaceutical Sciences, College of Pharmacy, Oregon State University, Corvallis, Oregon 97331, USA

[2] Department of Natural Products, Faculty of Pharmacy, King Abdulaziz University, Jeddah 21589, Kingdom of Saudi Arabia

[3] King Fahd Medical Research Center, King Abdulaziz University, Jeddah 21589, Kingdom of Saudi Arabia

[4] Department of Pharmacognosy, Faculty of Pharmacy, Suez Canal University, Ismailia 41522, Egypt

[5] Suez Canal University Hospitals, Suez Canal University, Ismailia 41522, Egypt

[6] Center for Marine Biotechnology and Biomedicine, Scripps Institution of Oceanography, University of California San Diego, La Jolla, California 92093, USA

[7] Skaggs School of Pharmacy and Pharmaceutical Sciences, University of California San Diego, La Jolla, California 92093, USA

E-mail: jane.ishmael@oregonstate.edu and kerry.mcphail@pharm.utah.edu

Table of Contents

Page 3	Figure S1<br>Partial ^{1}H NMR spectra (700 MHz, $CDCl_3$) for the α–proton region of DY-71-7 subfractions C (1-3).
Page 4	Figure S2<br>$LCMS^1$ spectra for subfraction D71-7 C1C reveals a product with the same m/z as hoiamide A.
Page 5	Figure S3<br>$LCMS^1$ spectra from dual injection of DY-71-7 subfraction C1C and authentic hoiamide A reveals matched elution times.
Page 6	Figure S4<br>Molecular network generated from $LCMS^2$ data after dual injection of DY-71-7 C1C and authentic hoiamide A.
Page 7	Figure S5.<br>Immunoblot analysis of α- and βIII-Tubulin expression in whole cell lysates prepared undifferentiated and differentiated human SH-SY5Y neuroblastoma cells.
Page 8	Figure S6<br>Comparative analysis of Gaussia luciferase (GLuc) expression and the viability of GLuc-U87 cells treated with rotenone in the presence, or absence, of N-acetylcysteine.
Page 9	Figure S7<br>Summary of workflow for separation and isolation of hoiamide A from a bioactive Red Sea cyanobacterial parent fraction (D71-7).
Page 10-12	Table S1<br>Comparative analysis of $LCMS^2$ data from dual injection of DY-71-7 subfraction C1C and authentic hoiamide A.
Page 13	Table S2<br>Comparative analysis of isotopic patterns of the hoiamide A substructure.
Page 14	Table S3<br>Primer sequences used for qPCR analysis.
Page 15-16	Experimental Section<br>Semi-purification of DY-71-7 extract
Page 17	References

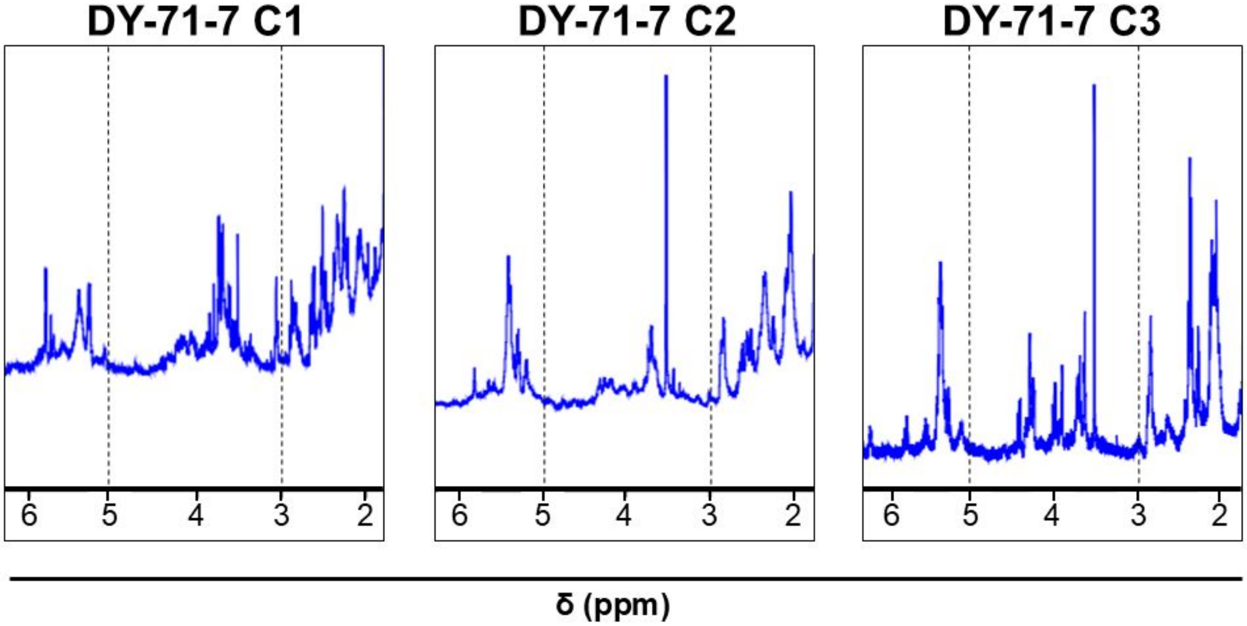

Figure S1. Partial ^{1}H NMR spectra (700 MHz, $CDCl_3$) for the α–proton region of DY-71-7 subfractions C1-3. Normal phase followed by reverse phase solid phase extraction (SPE) was used to isolate the less polar fractions of DY-71-7. ^{1}H NMR data were acquired on a Bruker 700 MHz NMR spectrometer. The acquired NMR data was interrogated for the presence of peaks in the 3-5 ppm region, indicating the presence of peptides.

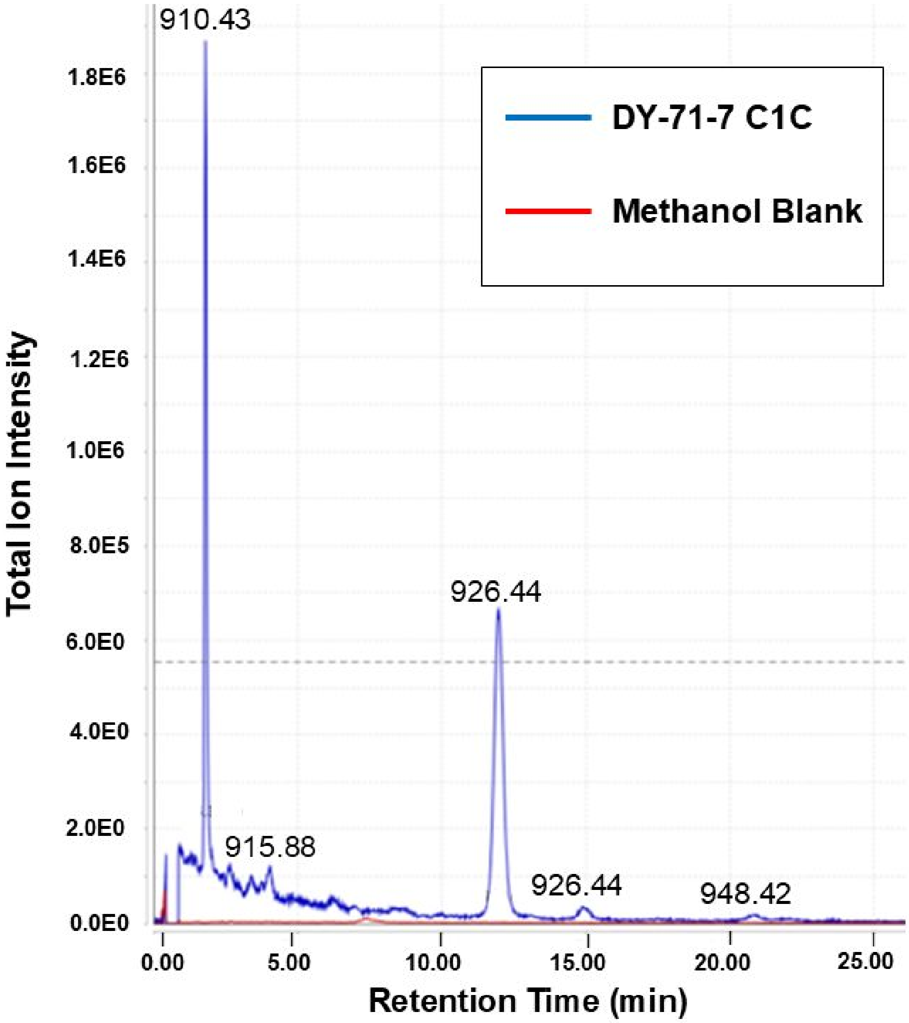

Figure S2. LCMS[1] spectrum for subfraction DY-71-7 C1C reveals a product with the same m/z as hoiamide A. Semi-preparative HPLC was used to further fractionate subfraction D71-7-C1C using a gradient method with a solvent composition of 15% acetonitrile in water + 1% formic acid to 100% acetonitrile + 1% formic acid. The LCMS[1] spectra from the fraction collected in 65% acetonitrile in water + 1% formic acid revealed a hoiamide-like m/z value at 926.4439.

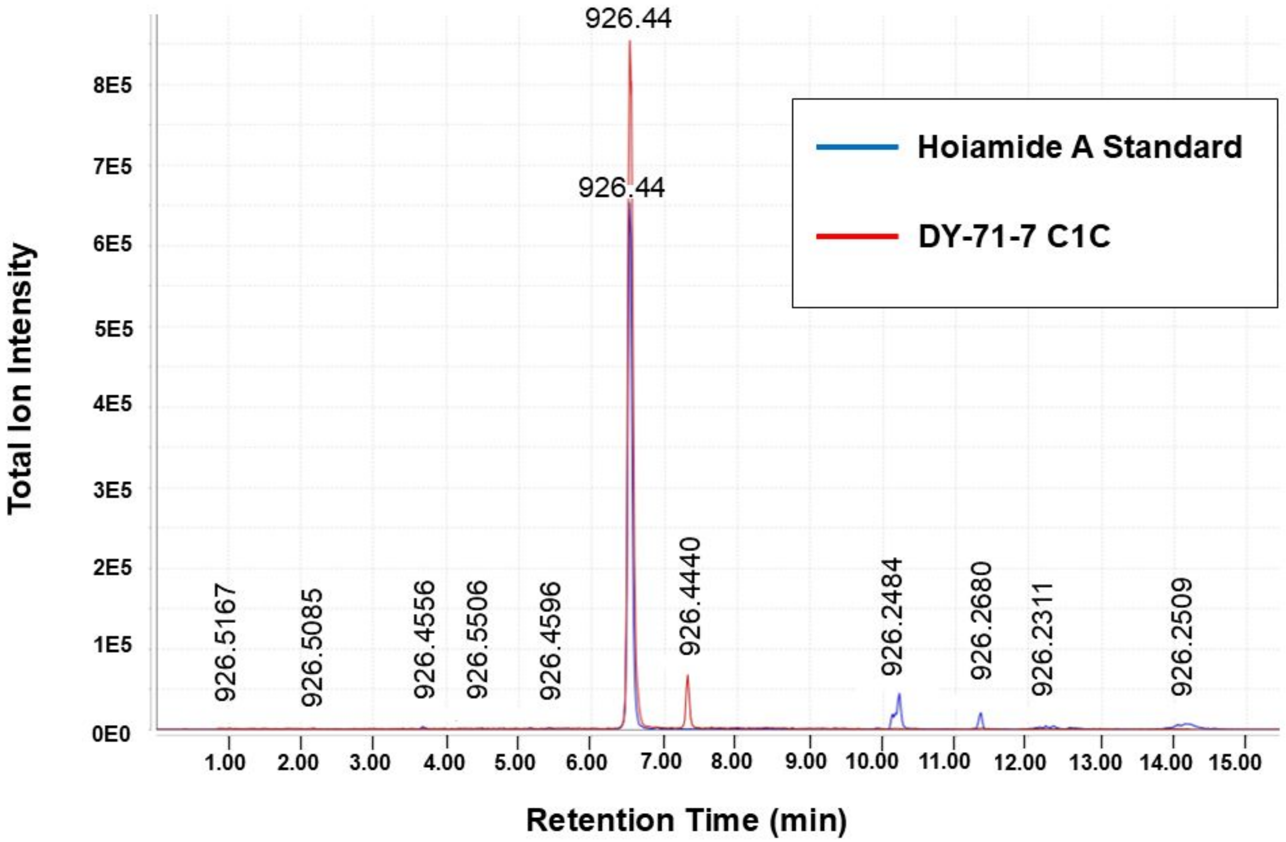

Figure S3. Dual LCMS[1] spectra for DY-71-7 subfraction C1C and authentic hoiamide A reveals matched elution times. LCMS[1] data from a dual injection assay with DY-71-7 C1C and a hoiamide A (**1**) authentic standard shows a matched elution time between the two compounds (t = 6.48 minutes). The m/z 926.44 peak identified in the C1C fraction matched that of a m/z 926.44 peak detected for authentic 1.

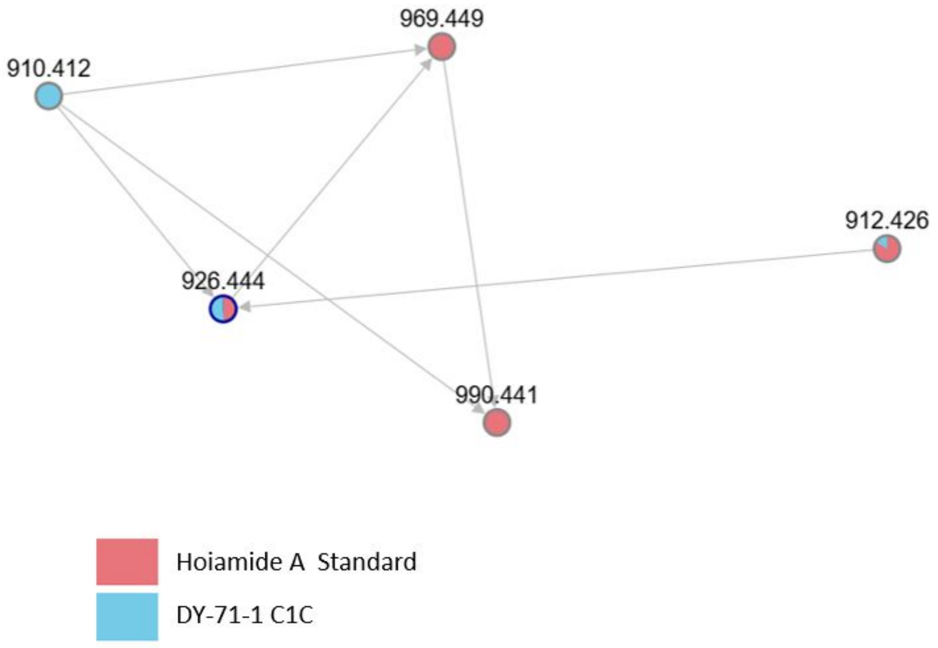

Figure S4. Molecular network generated from $LCMS^2$ data after dual-injection of DY-71-7 C1C and authentic hoiamide A. A classic molecular network was generated using analysis tools available through GNPS software. [1] $LCMS^2$ data from a dual-injection experiment was used to compare spectra from the DY-71-7 C1C subfraction (blue) and the hoiamide A standard (pink). Nodes represent individual mass features; a node (926.444) consistent with the mass of hoiamide A is present in both samples. Other nodes suggest the presence of adducts, or potentially hoiamide analogues.

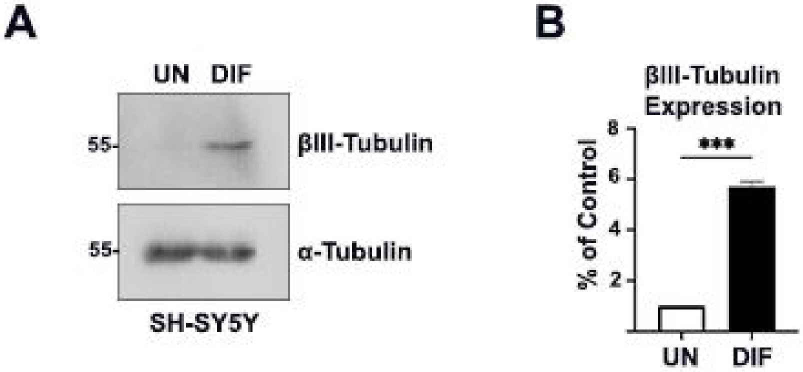

Figure S5. Immunoblot analysis of α-tubulin and βIII-tubulin expression in whole cell lysates prepared from undifferentiated and differentiated human SH-SY5Y neuroblastoma cells. Expression of microtubule isoforms in undifferentiated and differentiated human SH-SY5Y neuroblastoma cells. SH-SY5Y cells were terminally differentiated over 11 days by sequential treatment with retinoic acid (10 μM) for five days, followed by brain-derived neurotrophic factor (BDNF; 50 ng/mL) for five days. [2] (A) Lysates from undifferentiated control cultures and differentiated SH-SY5Y cultures were processed for Western blot analysis of α-tubulin and βIII-tubulin expression. (B) Histogram represents quantification of βIII-tubulin expression (mean ± S.E.) in differentiated cells, relative to undifferentiated cells, from two independent cultures. Statistical significance of change is: *** $p < 0.005$.

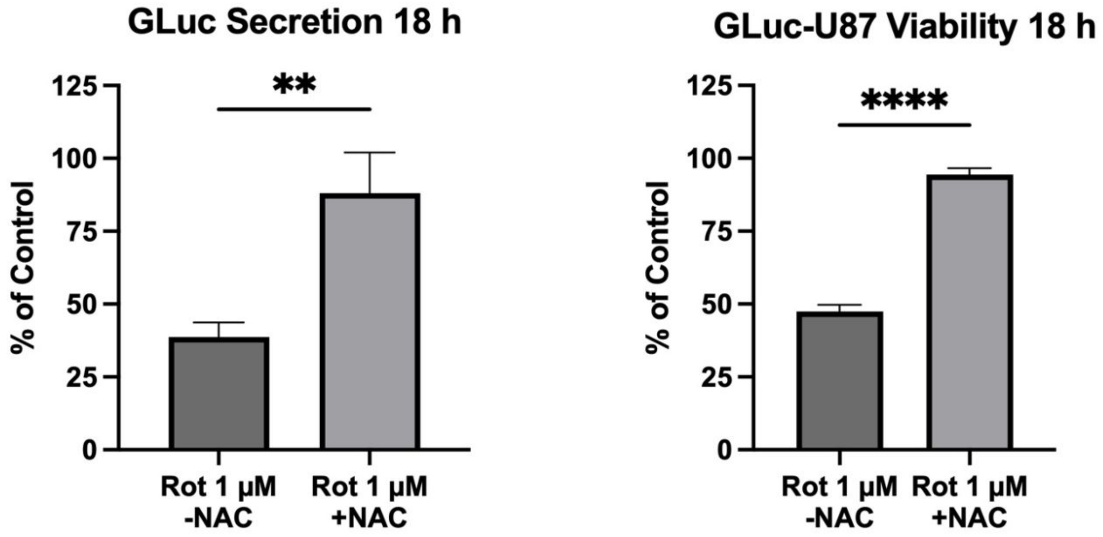

Figure S6. Comparative analysis of Gaussia luciferase (GLuc) expression and the viability of GLuc-U87 cells treated with rotenone in the presence, or absence, of N-acetylcysteine. Comparative analysis of the secretory function and viability of rotenone-treated cells in the presence or absence of N-acetylcysteine (NAC). The secretory function and viability of human U87-MG glioblastoma cells expressing Gaussia luciferase (GLuc) was assessed 18 h after treatment with rotenone (Rot; 1 µM), or vehicle (0.1% DMSO) in the presence, and absence of NAC (3 mM). Data are expressed as average ± S.E. (n=3 wells) relative to solvent vehicle (100%) from an experiment that was repeated at least three times with similar results determinations. Statistical significance of change is: ** $p < 0.01$, **** $p < 0.001$.

Figure S7. Summary of workflow for separation and isolation of hoiamide A from a bioactive Red Sea cyanobacterial parent fraction (D71-7).

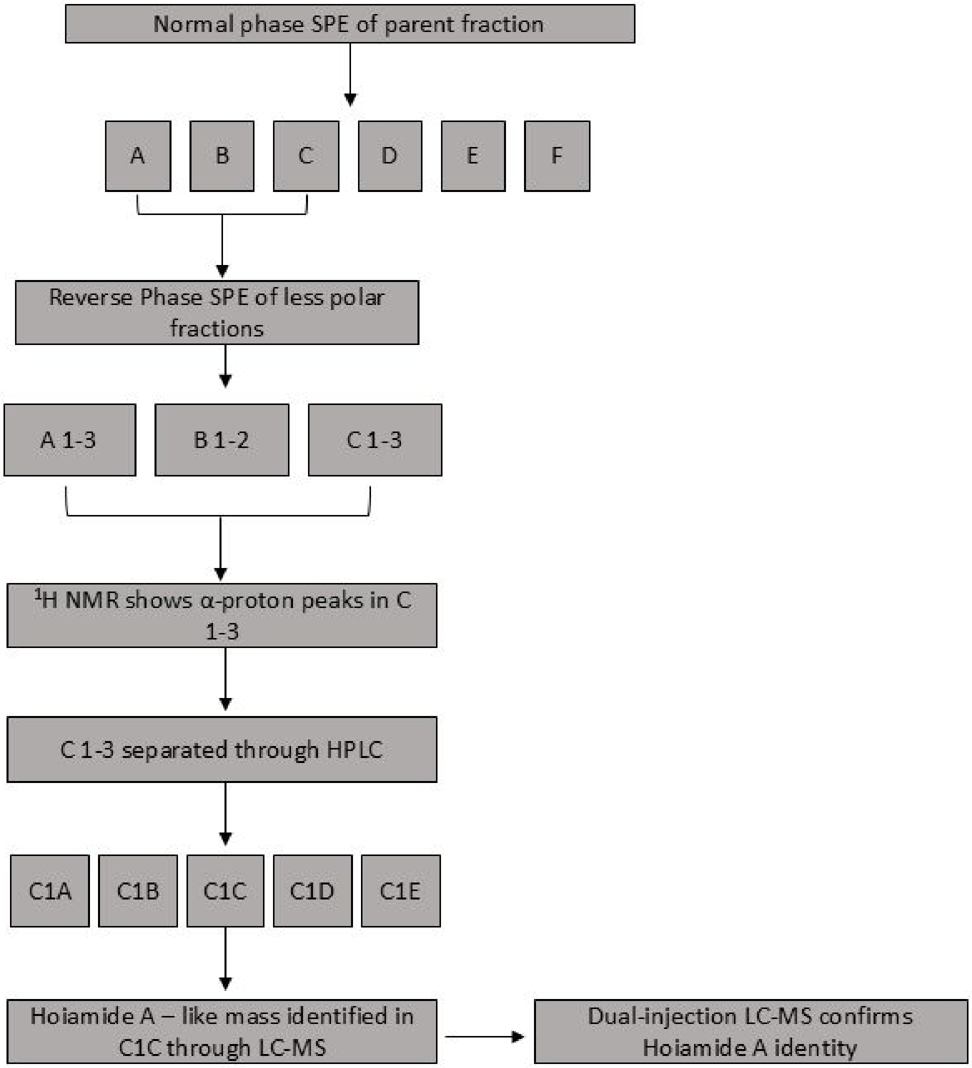

Table S1 Comparative analysis of LCMS^2 data from dual injection of DY-71-7 C1C subfraction and authentic hoiamide A.

DY-71-7 C1C		Hoiamide A	
m/z	Intensity	m/z	Intensity
55.054	154.128	55.054	76.276
57.056	90.894	57.054	142.311
69.07	1423.57	69.07	721.892
-	-	71.085	82.781
85.066	296.162	85.064	188.533
100.022	732.592	100.024	381.341
120.065	296.245	-	-
154.122	819.457	154.122	479.925
163.147	1193.08	163.147	595.728
172.034	1770.93	172.033	1052.66
181.157	54.124	181.157	110.54
-	-	184.074	135.231
197.019	252.957	-	-
217.056	143.562	-	-
239.029	729.068	239.03	344.857
265.069	332.61	-	-
277.081	621.761	277.083	414.373
281.131	569.059	281.131	366.02
299.141	349.742	299.141	323.527
-	-	309.107	151.206
359.16	1218.64	359.16	827.171
360.164	290.181	-	-
377.17	1521	377.171	1264.49
378.172	226.618	378.175	137.716

-	-	383.07	161.636
409.175	795.833	409.197	896.673
421.116	190.815	421.116	243.367
458.183	315.197	458.178	183.567
475.218	192.589	475.219	95.312
503.194	771.455	503.194	673.257
-	-	508.211	233.565
521.208	527.45	521.205	567.367
535.22	215.97	-	-
553.233	676.439	553.232	1128.5
-	-	554.233	188.993
575.222	156.952	575.216	248.669
-	-	601.266	87.769
613.303	123.988	613.303	216.308
-	-	651.304	169.108
657.294	311.076	657.292	532.994
663.342	206.695	663.341	507.662
675.305	173.592	675.305	297.09
689.319	423.801	689.32	604.458
707.331	687.663	707.332	1162.07
708.332	218.55	708.334	432.578
756.274	628.996	756.275	1428.68
757.275	116.889	757.276	355.125
794.326	974.109	794.327	2325.13
795.329	440.961	795.329	925.752
-	-	796.326	347.009
814.328	207.246	814.32	183.692
826.353	396.848	826.352	975.381
876.404	1813.27	876.405	3638.4
877.405	636.348	877.408	1367.43
880.442	70.766	-	-

908.43	3540.42	908.431	7645.68
909.434	1472.41	909.434	2613.12

Table S2 Comparative analysis of isotopic patterns of the hoiamide A substructure.

Authentic Hoiamide A [3]		DY-71-7 C1C	
Peak m/z	Intensity (% of parent mass peak)	Peak m/z	Intensity (% of parent mass peak)
926	100	926.44	100
927	58	927.44	64.56
928	28	928.44	34.07
929	10	929.44	8.55
930	3	930.44	1.33

Table S3 Primer sequences used for qPCR analysis

GENE	Forward Primer	Reverse Primer
HSPA5 BiP GRP78	CATCACGCCGTCCTATGTCG	CGTCAAAGACCGTGTTCTCG
CHOP	GGAAACAGAGTGGTCATTCCC	CTGCTTGAGCCGTTCATTCTC
sXBP1	GGTCTGCTGAGTCCGCAGCAG G	GGGCTTGGTATATATGTGG
HIF1A	CCTCTGGACTTGCCTTTCCT	CGACGTTCAGAACTTATCTTTTTCT
\NRF2	AGGTTGCCCACATTCCCAAA	ACGTAGCCGAAGAAACCTCA
GPX1	TATCGAGAATGTGGCGTCCC	TCTTGGCGTTCTCCTGATGC
GPX4	TGGACGAGGGGAGGAGC	GGGACGCGCACATGGT
GSR	ACTACCTGGTGATCGGGGG	ACATCCAACATTCACGCAAGTG
SOD1	AGGCATGTTGGAGACTTGGG	TGCTTTTTCATGGACCACCAG
β-actin	AGGCACCAGGGCGTGAT	GCCCACATAGGAATCCTTCTGAC

Experimental Procedures for Isolation of Hoiamide A from Red Sea Cyanobacteria

Chemicals and Reagents

HPLC-grade 100% hexanes were from JT Baker (Phillipsburg, NJ, USA). HPLC-grade ethyl acetate was from VWR (Radnor, PA, USA). LCMS-grade methanol was from Honeywell – Burdick and Jackson (Muskegon, MI, USA). LCMS grade acetonitrile was from OmniSolv (Aston, PA, USA). Deuterated chloroform was from Cambridge Isotope Laboratories (Tewksbury, MA, USA). Formic acid, HPLC grade methanol and HPLC-grade acetonitrile were from Thermo Fisher Scientific (Waltham, MA, USA).

Solid Phase Extraction

Normal phase solid phase extraction (SPE) was used to collect the initial fractions of DY-71-7. A silica column was primed with a wash of 100% hexanes followed by 40% ethyl acetate in hexanes, 80% ethyl acetate in hexanes, 100% ethyl acetate, and finally 25% methanol in ethyl acetate. Samples were dissolved in 40% ethyl acetate in hexanes. Fraction A and B were collected in 40% ethyl acetate in hexanes. Fraction C was collected in 80% ethyl acetate in hexanes. Fraction D was collected in 100% ethyl acetate. Fraction E was collected in 25% methanol in ethyl acetate. Finally, fraction F was collected in 100% methanol.

Reverse phase SPE was used to further fractionate DY-71-7 A-C. Because more than 10 mg of fractions A and C were isolated during normal phase SPE, reverse phase SPE was performed using a 500mg C18 column. The column was primed with 100% acetonitrile, then equilibrated using 75% acetonitrile in Mili-Q water. Fraction 1 of DY-71-7 A and C was collected in 75% acetonitrile in water. Fraction 2 was collected in 100% acetonitrile. Finally, fraction 3 was collected as a dichloromethane wash of the column. Less than 10 mg of fraction B was isolated from normal phase SPE, so a 100 mg C18 column was used. The column was primed with 100% acetonitrile, then equilibrated with 100% acetonitrile. Fraction 1 of DY-71-7 B was collected in 100% acetonitrile. Fraction 2 was collected as a dichloromethane wash of the column. All solvents used in SPE were HPLC-grade.

Liquid Chromatography Mass Spectrometry (LCMS)

Liquid Chromatography – Mass Spectrometry (LCMS) data was collected on an Agilent 6545 Q-TOF mass spectrometer equipped with an Agilent 1260 Infinity II HPLC system Samples were prepared at a concentration of 0.1 mg/mL in LCMS-grade methanol. Samples were filtered through syringe filter with a 0.2 µM PTFE membrane (VWR, Radnor, PA, USA) before being added to a 300 µL 6x29 mm

autosampler vial (MicroSolv Technology Corporation, Leland, NC) and placed inside a 9 mm amber LCMS vial (VWR, Radnor, PA, USA). Samples were separated using a gradient method of 50% LCMS grade acetonitrile in LCMS grade water + 1% formic acid to 100% LCMS grade acetonitrile + 1% formic acid with an injection volume of 5 µL and a flow rate of 0.4 mL/min. Separation was performed using a Phenomenex Kinetix 2.6 µm C18 column (100 Angstroms, 50 x 2.1 mm, Phenomenex, Torrance, CA, USA). The column compartment was maintained at 30 °C. Data-dependent acquisition of MS^2 spectra was performed in positive mode. Electrospray ionization parameters were set to a gas temperature of 325 °C, a gas flow of 10 L/min, a nebulizer 20 psi, a sheath gas temperature of 375 °C, and a sheath gas flow of 12 L/min. The spray voltage was set to 600 V. MS scan range was set to m/z 50−3,000 and the scan rate was 10 spectra/s. Collision energy was set to a stepwise increase from 20 to 40 to 60 eV. MS scans were selected when precursor counts reached 1,000 counts and spectra were excluded after six were collected. For $LCMS^2$ data processing, raw spectra were converted to .mzML files using MSconvert (ProteoWizard). MS feature extraction was performed using MZmine 3.0. The feature table .csv and .mgf files were exported and uploaded to GNPS (gnps.ucsd.edu) for feature-based molecular networking (FBMN). Molecular networks were visualized with Cytoscape.

Nuclear Magnetic Resonance (NMR) Spectroscopy

Samples DY-71-7 A(1-3), B(1-2), and C(1-3) were prepared by dissolving the fractions into $CDCl_3$. NMR tubes were from Wilmad (MHz, 300 MHz, OD: 5mm, length 7". Vineland, NJ, USA). Data was collected on a Bruker 700 MHz NMR and analyzed using TopSpin software. Spectra for each fraction were searched for alpha proton signals in the 3-5 ppm region.

High Performance Liquid Chromatography (HPLC)

Samples were prepared with 10 mg of each fraction (DY-71-7 C1-3) per 1 mL of HPLC-grade methanol. The samples were analyzed using a Shimadzu HPLC (FRC-10A Fraction Collector, CMB-20A Communications Bus Module, DGU-20A5 Degasser, LC-20AD Liquid Chromatograph). Samples were separated using a Phenomenex Synergi 4µ Hydro-RP column (80 Angstroms, 250 x 4.60 mm, 4 µ micron). A gradient method with an injection volume of 0.8mL/min and a solvent composition of 15% acetonitrile in water + 1% formic acid to 100% acetonitrile + 1% formic acid was used to separate the sample over 35 minutes. Subfractions A-E were collected from fractions DY-71-7 C1-3. A was collected from 0-5 minutes in 15% acetonitrile. B was collected from 5-15 minutes during a 15-65% acetonitrile gradient. C was collected from 15-25 minutes during an isocratic 65% acetonitrile period in the method.

D was collected from 25-30 minutes during a 65-100% acetonitrile gradient E was collected from 30-35 minutes during a 100% acetonitrile wash of the column.

References

1. Wang, M.; Carver, J. J.; Phelan, V. V.; Sanchez, L. M.; Garg, N.; Peng, Y.; Nguyen, D. D.; Watrous, J.; Kapono, C. A.; Luzzatto-Knaan, T.; Porto, C.; Bouslimani, A.; Melnik, A. V.; Meehan, M. J.; Liu, W. T.; Crusemann, M.; Boudreau, P. D.; Esquenazi, E.; Sandoval-Calderon, M.; Kersten, R. D.; Pace, L. A.; Quinn, R. A.; Duncan, K. R.; Hsu, C. C.; Floros, D. J.; Gavilan, R. G.; Kleigrewe, K.; Northen, T.; Dutton, R. J.; Parrot, D.; Carlson, E. E.; Aigle, B.; Michelsen, C. F.; Jelsbak, L.; Sohlenkamp, C.; Pevzner, P.; Edlund, A.; McLean, J.; Piel, J.; Murphy, B. T.; Gerwick, L.; Liaw, C. C.; Yang, Y. L.; Humpf, H. U.; Maansson, M.; Keyzers, R. A.; Sims, A. C.; Johnson, A. R.; Sidebottom, A. M.; Sedio, B. E.; Klitgaard, A.; Larson, C. B.; P, C. A. B.; Torres-Mendoza, D.; Gonzalez, D. J.; Silva, D. B.; Marques, L. M.; Demarque, D. P.; Pociute, E.; O'Neill, E. C.; Briand, E.; Helfrich, E. J. N.; Granatosky, E. A.; Glukhov, E.; Ryffel, F.; Houson, H.; Mohimani, H.; Kharbush, J. J.; Zeng, Y.; Vorholt, J. A.; Kurita, K. L.; Charusanti, P.; McPhail, K. L.; Nielsen, K. F.; Vuong, L.; Elfeki, M.; Traxler, M. F.; Engene, N.; Koyama, N.; Vining, O. B.; Baric, R.; Silva, R. R.; Mascuch, S. J.; Tomasi, S.; Jenkins, S.; Macherla, V.; Hoffman, T.; Agarwal, V.; Williams, P. G.; Dai, J.; Neupane, R.; Gurr, J.; Rodriguez, A. M. C.; Lamsa, A.; Zhang, C.; Dorrestein, K.; Duggan, B. M.; Almaliti, J.; Allard, P. M.; Phapale, P.; Nothias, L. F.; Alexandrov, T.; Litaudon, M.; Wolfender, J. L.; Kyle, J. E.; Metz, T. O.; Peryea, T.; Nguyen, D. T.; VanLeer, D.; Shinn, P.; Jadhav, A.; Muller, R.; Waters, K. M.; Shi, W.; Liu, X.; Zhang, L.; Knight, R.; Jensen, P. R.; Palsson, B. O.; Pogliano, K.; Linington, R. G.; Gutierrez, M.; Lopes, N. P.; Gerwick, W. H.; Moore, B. S.; Dorrestein, P. C.; Bandeira, N., Sharing and community curation of mass spectrometry data with Global Natural Products Social Molecular Networking. Nat Biotechnol 2016, 34 (8), 828-837.

2. Dravid, A.; Raos, B.; Svirskis, D.; O'Carroll, S. J., Optimised techniques for high-throughput screening of differentiated SH-SY5Y cells and application for neurite outgrowth assays. Sci Rep 2021, 11 (1), 23935.

3. Pereira, A.; Cao, Z.; Murray, T. F.; Gerwick, W. H., Hoiamide a, a sodium channel activator of unusual architecture from a consortium of two papua new Guinea cyanobacteria. Chem Biol 2009, 16 (8), 893-906.
